# Association between polypharmacy and chronic kidney disease among community-dwelling older people: a longitudinal study in southern China

**DOI:** 10.1186/s12882-024-03606-x

**Published:** 2024-05-17

**Authors:** Bowen Zhang, Jingrui Wang, Nan Liu, Weijia Liu, Ruihan Xi, Peixi Wang

**Affiliations:** 1 General Practice Center, The seventh Affiliated Hospital of Southern Medical University, Foshan, 528244 China; 2 School of Nursing and Health, Henan University, Kaifeng, 475004 China; 3 College of Public Health, Zhengzhou University, Zhengzhou, 540001 P. R. China; 4 Institute of Environment and Health, Health Science Center, South China Hospital, Shenzhen University, Shenzhen, 518116 P. R. China

**Keywords:** Chronic kidney disease, Multimorbidity patterns, Polypharmacy, Older people

## Abstract

**Background:**

Polypharmacy would increase the risk of adverse drug events and the burden of renal drug excretion among older people. Nevertheless, the association between the number of medication and the risk of chronic kidney disease (CKD) remains controversial. Therefore, this study aims to investigate the association between the number of medication and the incidence of CKD in older people.

**Methods:**

This study investigates the association between the number of medications and CKD in 2672 elderly people (≥ 65 years older) of the community health service center in southern China between 2019 and 2022. Logistic regression analysis was used to evaluate the relationship between polypharmacy and CKD.

**Results:**

At baseline, the average age of the study subjects was 71.86 ± 4.60, 61.2% were females, and 53 (2.0%) suffer from polypharmacy. During an average follow-up of 3 years, new-onset CKD developed in 413 (15.5%) participants. Logistic regression analysis revealed that taking a higher number of medications was associated with increase of CKD. Compared with people who didn’t take medication, a higher risk of CKD was observed in the older people who taken more than five medications (OR 3.731, 95% CI 1.988, 7.003), followed by those who take four (OR 1.621, 95% CI 1.041, 2.525), three (OR 1.696, 95% CI 1.178, 2.441), two drugs (OR 1.585, 95% CI 1.167, 2.153), or one drug (OR 1.503, 95% CI 1.097, 2.053). Furthermore, age, systolic blood pressure (SBP), white blood cell (WBC), blood urea nitrogen (BUN) and triglyceride (TG) were also independent risk factors CKD (*P* < 0.05).

**Conclusion:**

The number of medications was associated with CKD in older people. As the number of medications taken increased, the risk of CKD was increased.

**Supplementary Information:**

The online version contains supplementary material available at 10.1186/s12882-024-03606-x.

## Introduction

In recent years, with the increasing aging of the population, the burden of disease and the limited public health resources are bringing global concern seriously [1]. According to World Population Prospects, the number of people over 65 in the world will increase from 9% in 2019 to 16% in 2050 (https://www.un.org/zh/globalissues/ageing. Accessed 17 July 2023). Due to population aging and the extended life expectancy, the prevalence of comorbidity is growing, which inevitably leads to the use of polypharmacy [2]. Polypharmacy is the concomitant use of several medicines by a single individual [3]. According to a nationwide large sample survey, the prevalence of comorbidity was as high as 42.4% in the Chinese older population [4], and the number of medications increased by 0.95 accompanied by each additional chronic disease in older people [5]. Comorbidity has become a serious public health problem, resulting in substantial economic cost and health burden to the health system.

Due to more comorbidities, the medication use among elderly people is high. A prospective cohort study from Sweden showed that the prevalence of polypharmacy (≥ 5 medications) was around 44.0% among older adults [5]. Another cross-sectional study from the UK found that more than 24.0% of people were affected by polypharmacy [6]. In China, approximately 70% of older adults (≥ 80 years old) reported having polypharmacy (≥ 6 medications) [7]. This difference may be related to differences in the definition, geography, and population of polypharmacy, but the same high incidence rate cannot be ignored [8]. Despite the potential beneficial effect of combination medicines, medication use is associated with undesirable health outcomes. A medication used to treat a disease or symptom might have a negative effect on another disease or symptom [9]. Polypharmacy is closely related to drug-drug, drug-disease interactions and adverse drug reactions, which can lead to hospital admission, worsen chronic conditions and an increase in morbidity and mortality, especially in older people [10, 11]. The functional reserves of multiple systems and organs are damaged because of the aging process of humans, which will increase the sensitivity to medications [12]. Many systemic therapeutic drugs are metabolized in the kidney, therefore, polypharmacy has the potential to deteriorate kidney function. The incidence of chronic kidney disease (CKD) has increased worldwide, and it represents a major global public burden.

It is noteworthy that the older people need to use multiple medications in the treatment process due to the common existence of multiple diseases. That is to say, the elderly population at greater risk of polypharmacy and kidney damage. Nowadays, studies have found a link between polypharmacy and an increased risk of CKD [13–15]. A study reported that polypharmacy was associated with decreased estimated glomerular filtration rate (eGFR) in specific multimorbidity patterns for older people in Spain [16]. The risk of adverse drug reactions increases in older population due to polypharmacy (≥ 5 medications) as well as altered drug pharmacokinetics and pharmacodynamics caused by age-related changes, altered nutritional state, and reduced kidney clearance [17]. Currently, many studies used more than 5 drugs as polypharmacy to analyze the relationship between polypharmacy and potential adverse outcomes, which may underestimate the impact of polypharmacy on CKD. A 2-year prospective observational study on patients with knee osteoarthritis found that for every additional medication taken within 24 months, kidney function decreased by 0.39 mL/min/1.73 m^2^ eGFR [18]. Moreover, increased medication counts from the enrollment were associated with increased renal hazard events and cardiovascular events, and all-cause mortality in patients with CKD in some studies [15, 19, 20], which raises the possibility for a vicious cycle of the number of medications and worsening kidney condition.

Although studies of the association between polypharmacy and the higher risk of reduced kidney function, hospitalization, and death have confirmed [18, 21], only limited data is available on how the number of medications affects kidney function prospectively among community-dwelling adults aged 65 years and older in China. Exploring the relationship between the number of medications and CKD is of great significance for promoting rational drug use and rational utilization of medical resources for older people. Currently, few studies focus on the relationship between the number of medications and the new incidence rate of CKD in elderly population. Based on the above, using the data from the 2019–2022 period of southern Chinese communities, we performed a longitudinal study to analyze the relationship between medication use and CKD among older people.

## Materials and methods

### Participants

This is a cohort study of the elderly (≥ 65 years old) undergoing standardized management at a community health service center in the Pearl River Delta region of southern China. The baseline population consists of elderly individuals who received free health examinations in the community from January to September 2019. The health examination included self-reported medical history, physical examination, laboratory examination and instrument-based examination (abdominal ultra-sonography, electrocardiogram and chest X-ray). Individuals with a lack of necessary data (blood biochemical indicators) at baseline, CKD or severe kidney disease were excluded from the study, with 2767 individuals were included in the study and will undergo further health checks and follow-up in 2022. After a 3-year follow-up, of which 15 participants were excluded due to serum creatinine (Scr) deficiency and 80 participants were lost during follow-up. The final study sample included 2672 subjects. All procedures were carried out in accordance with the Declaration of Helsinki. This study was approved by the Ethics Committee of Henan University and all participants provided informed consent before the study.

### Data collection and measurements

#### Baseline examinations includes

Sociodemographic and health-related information including gender, age, the history of medication use, the history of chronic diseases, smoking and drinking history. The details (name, usage, dosage, and duration of use) of drugs will be documented, and the various drugs were classified according to the Anatomical Therapeutic Chemical classification system. According to the number of long-term medications taken (≥ 30 days) by the participants at baseline, they are divided into six categories (0, 1, 2, 3, 4, ≥ 5). Comorbidity is the coexistence of two or more chronic diseases or conditions. Smokers were defined as those who smoked one or more cigarettes a day for at least three months or had a history of smoking. Regular alcohol consumers were defined as those who consumed alcohol on average more than once a week within the last year. Defining regular exercise as maintaining at least 150–300 min of moderate intensity aerobic exercise or at least 75–150 min of high-intensity aerobic exercise per week for adults.

The physical examinations, including measurements of weight, height, systolic and diastolic blood pressure (SBP and DBP), were carried out by trained medical staff. Weight, height and waist circumference (WC) were measured with participants wearing light clothes and without shoes, and the body mass index (BMI) of the participants was calculated. After fasting for eight hours the previous night, blood samples were collected in the morning to test laboratory parameters that included alanine aminotransferase (ALT), aspartate aminotransferase (AST), total bilirubin (TBIL), serum creatinine (Scr), blood urea nitrogen (BUN), 24-hour urine protein, total cholesterol (TC), triglyceride (TG), low-density lipoprotein cholesterol (LDL-C), high-density lipoprotein cholesterol (HDL-C) and fasting plasma glucose (FPG). Venous blood samples were collected by trained medical staff, and analyzed with a blood analyzer (Mindray BC-2900, Shenzhen, China) and an automatic biochemical analyzer (Mindray BS-420, Shenzhen, China).

#### Follow-up and outcomes

The main outcome of follow-up is defined as CKD occurring during the annual examination in 2022. eGFR was calculated using serum creatinine, age, sex and race according to the Chronic Kidney Disease Epidemiology Collaboration (CKD-EPI) equation [22]. Specifically, CKD was considered when eGFR was < 60 mL/min/1.73 m^2^ or urinary protein was qualitatively positive.

### Statistical analyses

Statistical description and single factor analysis were conducted using SPSS 25.0 (SPSS Company, Chicago, Illinois), and logistic regression analysis was performed using R 4.1.3 software (https://www.r-project.org/. Accessed 28 November 2022) to calculate OR values. Continuous variables are presented as the mean ± standard deviation (SD) for normal distributions or the median (interquartile range) for non-normal distributions. Categorical variables are expressed as frequencies (percentages). For between-group comparisons, Student’s t-test (normal distribution) or Mann-Whitney U test (non-normal distribution) was used for continuous data, and the chi-square test for categorical variables. Moreover, logistic regression models were used to analyze the relationship between CKD and number of medications. Three models were created to analyze the association between different drug dosage groups and the incidence of CKD, and the significant indicators of univariate analysis (age, gender, systolic blood pressure, diastolic blood pressure, WC, BMI, WBC, BUN, TG, and LDL-C) and known risk factors of CKD(comorbidities)were included in the model for adjustment. The odds ratios (ORs) and 95% confidence intervals (CIs) were also calculated. The statistical significance level was set at α = 0.05 (two-tailed).

## Results

### Characteristics of study subjects

Table 1 shows the main baseline characteristics of the study population stratified by CKD status. A total of 2672 participants (1036 males and 1636 females) with an average age of 73.73 ± 6.39 years were included in this study. 1934 (72.4%) participants suffered comorbidity, 471 (17.6%) took two or more medications and 53 (2.0%) suffered from polypharmacy. On follow-up (mean of 3 years), new-onset CKD developed in 413 (15.5%) participants, and the prevalence of CKD in males was significantly higher than that in females (17.8% vs. 14.0%) (*P* < 0.05). Comparison between CKD and control groups indicated that the subjects with CKD were older, and high WBC, BUN, TG, SBP, DBP, WC, BMI and low LDL-C (*P* < 0.05). However, there was no significant such as comorbidity, marital status, smoking, drinking, physical activity, Hb, PLT, TC and HDL-C (*P* > 0.05). Specific data information was shown in Table 1.

**Table 1:** Characteristics of the participants with and without chronic kidney diseases at baseline

Variable	Total (*n*=2672)	Without CKD (*n*=2259)	With CKD (*n*=413)	t/x^2^/z	*P*
Age	71.86±4.60	71.49±4.37	73.90±5.24	−8.791	<0.001^a^
**Gender**				6.874	0.010^b^
Man	1036(38.77%)	852(37.72%)	184(44.55%)		
Woman	1636(61.23%)	1407(62.28%)	229(55.45%)		
**Education level**				1.341	0.496^b^
Primary school and below	2036(76.20%)	1713(75.83%)	323(78.21%)		
Junior and high school level	621(23.24%)	532(23.55%)	89(21.55%)		
College degree or above	15(0.56%)	14(0.62%)	1(0.24%)		
**Marital status**				1.07	0.303^b^
Married	2381(89.11%)	2019(89.38%)	362(87.65%)		
Unmarried/separated/widowed	291(10.89%)	240(10.62%)	51(12.35%)		
**Residence**				0.26	0.624^b^
Non-household	74(2.77%)	61(2.70%)	13(3.15%)		
Household	2598(97.23%)	2198(97.30%)	400(96.85%)		
Smoking (yes)	476(17.81%)	399(17.66%)	77(18.64%)	0.23	0.675^b^
Drinking (yes)	119(4.45%)	99(4.38%)	20(4.84%)	0.174	0.679^b^
**Physical activity (yes)**	1018(38.10%)	855(37.85%)	163(39.47%)	0.388	0.545^b^
SBP (mmHg)	133.03±15.08	132.43±15.50	136.28±17.06	−4.568	<0.001^a^
DBP (mmHg)	78.81±9.37	78.66±9.30	79.66±9.72	−1.993	0.046^a^
WC (cm)	86.01±9.34	85.68±9.34	87.85±9.15	−4.355	<0.001^a^
BMI (kg/m^2^)	24.52±3.59	24.42±3.57	25.08±3.68	−3.461	<0.001^a^
HB (g/L)	137.72±17.01	137.81±16.94	137.27±17.42	0.585	0.559 ^a^
PLT (10^9^/L)	255.29±63.55	255.24±63.22	258.14±67.31	0.087	0.930^a^
WBC (10^9^/L)	6.87±1.80	6.79±1.78	7.24±1.82	−4.702	<0.001^a^
ALT (U/L)	17.00(14.00, 22.00)	17.00(14.00, 22.00)	17.00(13.00, 23.00)	−0.72	0.472^c^
AST (U/L)	20.00(17.00, 23.00)	20.00(17.00, 23.00)	20.00(17.00, 24.00)	−1.815	0.070^c^
TBIL (mmol/L)	14.70(11.90, 17.88)	14.80(12.00, 17.90)	13.60(11.10, 17.60)	−3.118	0.002^c^
BUN (mmol/L)	5.70±2.34	5.61±2.45	6.19±1.52	−4.602	<0.001^a^
TC (mmol/L)	5.65±1.39	5.67±1.41	5.54±1.22	1.74	0.082^a^
TG (mmol/L)	1.55(1.10, 2.25)	1.52(1.09, 2.20)	1.73(1.22, 2.65)	−3.985	<0.001^c^
LDL-C (mmol/L)	2.92±0.85	2.94±0.85	2.82±0.86	2.593	0.010^a^
HDL-C (mmol/L)	1.61±0.56	1.61±0.43	1.62±1.01	−0.16	0.873^a^
**Comorbidities**				0.237	0.633^b^
Yes	1934(72.38%)	1631(72.20%)	303(73.37%)		
No	738(27.62%)	628(27.80%)	110(26.63%)		
**Polypharmacy**				17.208	<0.001^b^
Yes	53(1.98%)	34(1.50%)	19(4.60%)		
No	2619(98.02%)	2225(98.50%)	394(95.40%)		
**N. of medication**				52.895	<0.001^b^
0	1260(47.16%)	1124(49.76%)	136(32.93%)		
1	453(16.95%)	374(16.56%)	79(19.13%)		
2	471(17.63%)	382(16.91%)	89(21.55%)		
3	271(10.14%)	217(9.61%)	54(13.08%)		
4	164(6.14%)	128(5.67%)	36(8.72%)		
≥5	53(1.98%)	34(1.50%)	19(4.60%)		

*Abbreviations* “a” is t-value, “b” is *x*^2^-value, “c” is Z-value, CKD chronic kidney disease; SBP systolic blood pressure, DBP diastolic blood pressure, WC waist circumference, BMI body mass index, Hb hemoglobin, PLT platelet, WBC white blood cell, ALT alanine aminotransferase, AST aspartate aminotransferase, TBIL total bilirubin, BUN blood urea nitrogen, TC total cholesterol, TG triglyceride, LDL-C low-density lipoprotein cholesterol, HDL-C high-density lipoprotein cholesterol. HB: hemoglobin, PLT: platelet; WBC: white blood cell; ALT: alanine aminotransferase; AST: aspartate aminotransferase; BUN: blood urea nitrogen; TC: total cholesterol; TG: triglyceride; LDL-C: low-density lipoprotein cholesterol; HDL-C: high-density lipoprotein cholesterol

### The prevalences of diseases and polypharmacy

**Fig. 1:**
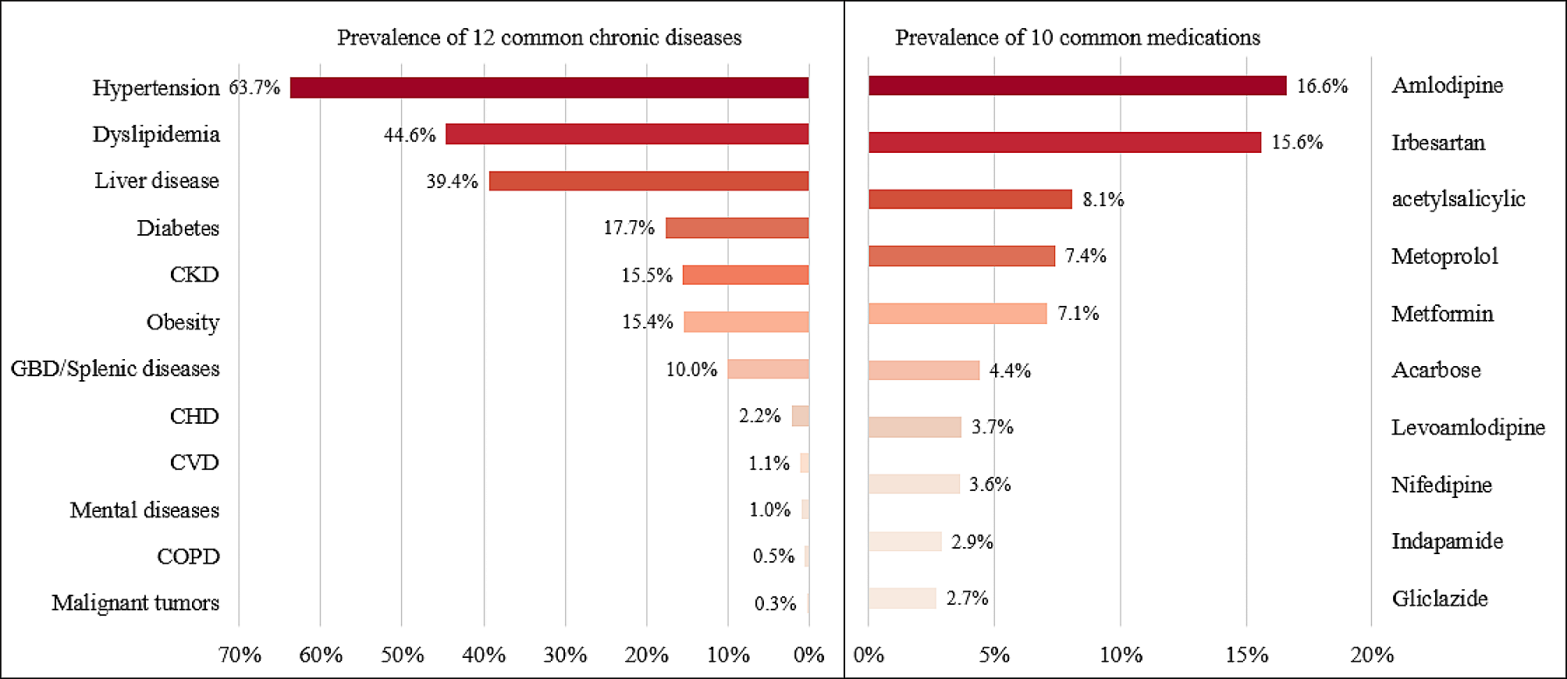
Prevalence of common chronic diseases and medications among the elderly people

**Fig. 2:**
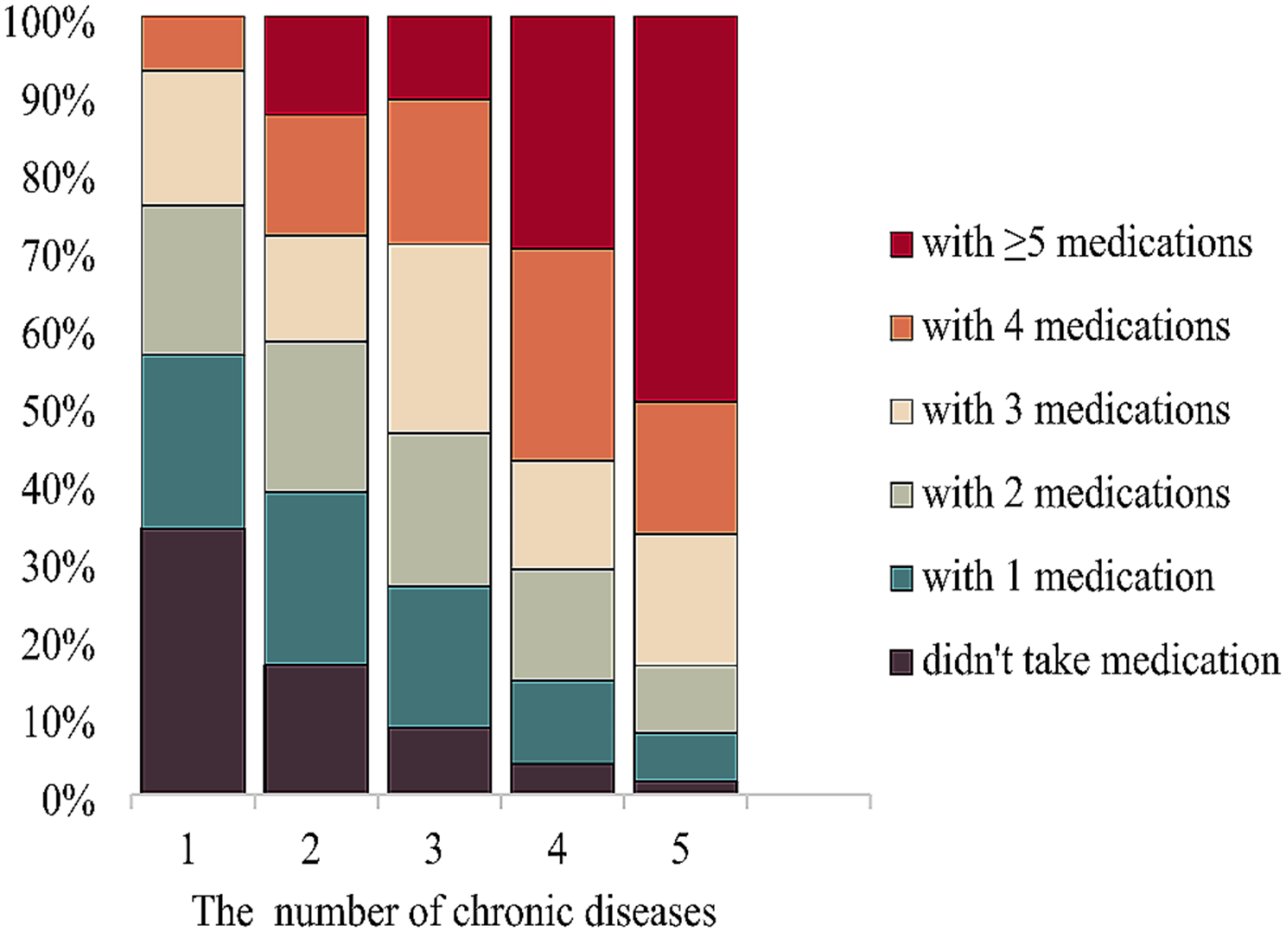
Relationship between the number of chronic diseases and the amount of medication used

Figure 1 shows the prevalence of common chronic diseases and medications among the elderly people. As Fig. 1 shown that the prevalence of hypertension (63.7%) was the highest, followed by dyslipidemia (44.6%), liver disease (39.4%), diabetes (17.7%) and chronic kidney disease (15.5%). The medications acting on the cardiovascular system were the most frequently used, along with the digestive and the metabolism system. The five most common drugs were amlodipine (16.6%), irbesartan (15.6%), acetylsalicylic (8.1%), metoprolol (7.4%), and metformin (7.1%). The percentage of medication use by the number of chronic diseases has shown in Fig. 2. Significantly, the higher the prevalence of polypharmacy in people with 5 or more chronic diseases (32.5%). As the number of chronic diseases increased, the amount of medication used was also increasing.

### The association between the number of medications and CKD

As shown in Table 2, our results demonstrated that people who took medications have an increased risk for CKD, compared with those who did not take medications after adjusting for potential confounders such as age, gender, SBP, DBP, WC, BMI, WBC, BUN, TG, LDL-C and comorbidities. Compared with those who did not take drugs, those who taken one medication (OR 1.503, 95% CI 1.097, 2.053), two medications (OR 1.585, 95% CI 1.167, 2.153), three medications (OR 1.671, CI 95% 1.158, 2.412), four medications (OR 1.621, 95% CI 1.041, 2.525), or more than five medications (OR 3.731; 95% CI 1.988, 7.003) have a higher risk of CKD. In addition, all analyses revealed that the risk of CKD significantly increased with age (OR: 1.112, 95% CI 1.088, 1.138) among elderly. We also observed that statistically significant between higher SBP (OR 1.012, 95% CI 1.004, 1.022), WBC (OR 1.111, 95% CI 1.047, 1.178), BUN (OR 1.081, 95% CI 1.033, 1.133) and TG (OR 1.106, 95% CI 1.035, 1.182) were the independent risk factors for CKD. In addition, we also found that compared to the population without medication use, the use of 6 (OR: 8.078, 95% CI 0.991, 65.808), 7(OR: 3.336, 95% CI 0.571, 21.504), and 8 drugs (OR: 12.896, 95% CI 0.787, 211.311) increased the risk of CKD, but there was no significant difference (*p* > 0.05). For more details, see Supplementary Table 1.

**Table 2:** The relationship between CKD and the number of medications in logistic regression model

Variable	B	${S_{\bar x}}$	Waldχ^2^	OR 95%CI	*P*
**Molde 1**					
Age	0.016	0.011	85.192	1.112(1.087–1.137)	<0.001
Gender (female)	−0.213	0.119	3.184	0.808(0.640–1.041)	0.074
Comorbidities (Yes)	−0.228	0.137	2.774	0.796(0.608–1.041)	0.096
SBP (mmHg)	0.012	0.005	7.417	1.012(1.003–1.022)	0.006
DBP (mmHg)	0.001	0.008	0.005	1.001(0.985–1.016)	0.941
WC (cm)	0.001	0.011	0.004	1.001(0.979–1.023)	0.952
BMI (kg/m^2^)	0.049	0.028	3.083	1.051(0.994–1.110)	0.079
WBC (10^9^/L)	0.104	0.03	11.92	1.109(1.046–1.177)	<0.001
TG (mmol/L)	0.098	0.034	8.33	1.103(1.032–1.179)	0.004
LDL-C (mmol/L)	−0.09	0.069	1.674	0.914(0.798–1.047)	0.196
BUN (mmol/L)	0.078	0.024	10.998	1.081(1.032–1.132)	0.001
TBIL (mmol/L)	−0.007	0.01	0.486	0.993(0.974–1.012)	0.486
N. of medication			24.502		<0.001
0				Reference	
1	0.409	0.16	6.513	1.506(1.100–2.062)	0.011
2	0.459	0.156	8.623	1.582(1.165–2.149)	0.003
3	0.525	0.186	7.976	1.691(1.174–2.435)	0.005
4	0.485	0.226	4.609	1.625(1.043–2.531)	0.032
≥5	1.311	0.321	16.61	3.710(1.975–6.969)	<0.001

Adjusted for age, gender, comorbidities, SBP, DBP, WC, BMI, WBC, BUN, TG, LDL-C, TBIL

*Abbreviations* SBP systolic blood pressure, DBP diastolic blood pressure, WC waist circumference, BMI body mass index, WBC: white blood cell, TG: triglyceride, LDL-C: low-density lipoprotein cholesterol, BUN: blood urea nitrogen, TBIL: total bilirubin

Because most elderly people take at least one medication, we further divided the population into separate groups based on whether they took ≥ 1 medication or ≥ 2 medications as controls. We found that as the number of medications increased, the risk of CKD also increased. Compared with the group taking ≥ 1 medication, the risk of developing CKD increased by 1.381, 1.477, and 3.223 times for those taking 2, 3, and ≥ 5 medications, respectively. Compared with the group taking ≥ 2 medications, the risk of developing CKD for those taking ≥ 5 medications still increased by 2.915 times (in Table 3).

**Table 3:** The relationship between CKD and the number of medications

Variable	B	${S_{\bar x}}$	Waldχ^2^	OR 95%CI	*P*
**Molde 2**					
N. of medication					
≥1			18.611	Reference	0.001
2	0.321	0.145	4.892	1.378(1.037-1.830)	0.027
3	0.387	0.177	4.796	1.472(1.041-2.082)	0.029
4	0.34	0.218	2.433	1.405(0.916-2.154)	0.119
≥5	1.164	0.316	13.572	3.203(1.724-5.949)	<0.001
**Molde 3**					
N. of medication					
≥2			14.123	Reference	0.003
3	0.297	0.171	2.997	1.345(0.962-1.883)	0.083
4	0.243	0.213	1.303	1.276(0.840-1.938)	0.254
≥5	1.064	0.312	11.576	2.897(1.570-5.348)	0.001

Adjusted for age, gender, SBP, DBP, WC, BMI, WBC, BUN, TG, LDL-C, comorbidities

*Abbreviations* SBP systolic blood pressure, DBP diastolic blood pressure, WC waist circumference, BMI body mass index, WBC: white blood cell, TG: triglyceride, LDL-C: low-density lipoprotein cholesterol, BUN: blood urea nitrogen

## Discussion

### Main findings

**Fig. 3:**
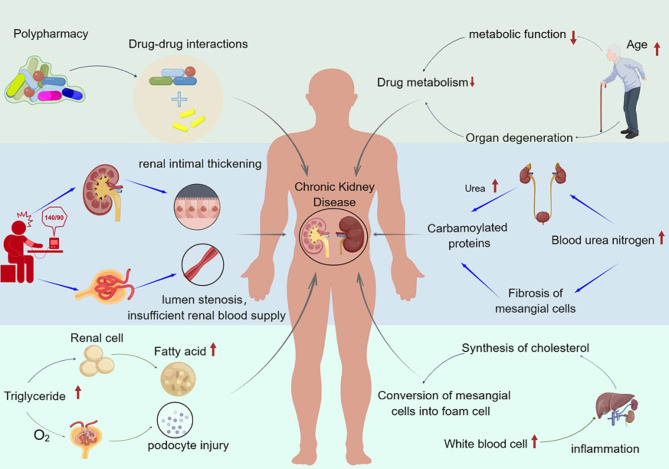
The risk factors and related influencing mechanisms of chronic kidney disease (This figure was drawn by Figdraw)

To our knowledge, our study was the first to apply longitudinal analysis to investigate the relationship between the risk of CKD and the number of medications among elderly people in China. According to the World Health Organization (WHO), kidney diseases were the 10th leading cause of death worldwide in 2019 [23]. Therefore, it is urgent to identify the potential risk factors of CKD to reduce the disease burden and mortality of patients. In this elderly sample from southern China, an average follow-up of 3 years, new-onset CKD developed in 413 (15.5%) participants. After adjusting for multiple confounding factors, the risk of CKD still increases with the increase of drug quantity. In addition, we also found that age, SBP, WBC, TG, and BUN being independent risk factors for CKD. The risk factors and related influencing mechanisms of chronic kidney disease are shown in Fig. 3.

### Comparison with previous studies

Polypharmacy for the elderly is a global problem, and the situation is constantly deteriorating. Due to the fact that elderly people often suffer from more diseases, they need to receive more medication treatment. A study found that the overall proportion of elderly people in the United States using multiple medications continues to rise, from 23.5% in 1999–2000 to 44.1% in 2017-2018 [24]. In this study, the prevalence of polypharmacy (2.0%, ≥ 5 medications) in southern China was lower than that in other regions (14.2%) in Chinese communities [25], but similar to Chen’ s study (2.1%, ≥ 5 medications) [26]. Polypharmacy not only reflects poor health status but the number and reflects severity of comorbidities [27]. In our study, we also found that as the number of chronic diseases in the elderly increases, the prevalence of polypharmacy also increases.

Polypharmacy has been linked to numerous negative clinical outcomes such as hospitalization, fragility fracture, kidney damage, and even mortality [15, 21, 28]. A cross-sectional study found that polypharmacy (≥ 5 medications) was associated with an increased risk of CKD after multivariate adjusted analysis in elderly people (≥ 60 years) in Germany [29]. Similarly, a study found that polypharmacy (≥ 5 medications) in cardiovascular outpatient patients is not only related to renal dysfunction, but also accelerates the deterioration of renal function [30]. Different from previous researched, we explored the relationship between the risk of CKD and the number of medications among elderly people. Significantly, after adjusting for confounders, the number of medications was still associated with CKD in older people. Compared to individuals who did not use medication, not only were taking ≥ 5 medications a contributing factor to the occurrence of CKD, but those who took 1, 2, 3, and 4 medications also had an increased risk of CKD by 1.503, 1.585, 1.671, and 1.162 times. And the risk of CKD increased with a higher number of medications in older people. In addition, a study also found that for every additional medication taken within 2 years, renal function decreased by 0.39 mL/min/1.73 m2 eGFR in patients with knee osteoarthritis [18]. Although most studies have used more than 5 drugs as polypharmacy, there is currently no clear international standard [31–33], and using this standard alone may underestimate the impact of polypharmacy on CKD. Therefore, clinical healthcare providers should strictly manage the medication usage of the elderly population to avoid the renal burden caused by unnecessary medication use.

Although the design of our study cannot explain the mechanism of the impact between CKD and the number of drugs used, we reasonably assume that they may stem from the patient’s poor baseline health status and comorbidity, deterioration of renal function with aging, drug-drug interactions (DDIs) and inappropriate prescriptions [16, 34, 35]. It is generally believed that the function of metabolism and clearance of medications might be decreased with aging, leading to older people being more vulnerable to damage. Due to pathological changes in organs, the impact of drugs of similar concentrations on the site of action may be greater or smaller in elderly people than in young people [36]. Along with aging, liver and kidney function will deteriorate, affecting the drug metabolism ability of elderly patients [37, 38]. Given these changes, elderly people are more sensitive to many medications and are also more sensitive to changes in medication. In the present study, we also observed an independent positive association between age and CKD risk in Chinese elderly population. Therefore, it is recommended to assess kidney function and adjust drug use for older people according to their drug metabolism.

Studies have found that polypharmacy is a key risk factor for DDIs and the risk of potential DDIs increases exponentially with the number of medications used [39, 40]. DDIs refers to the change in drug efficacy caused by the simultaneous addition of another drug to treat the same or different diseases. Simultaneously, some researches has found that kidney damage from polypharmacy can be caused by the increased risk of DDIs [15, 41]. This group not only has a high incidence rate of CKD patients, but also has a high burden of chronic complications, often using multiple drugs. Research has found that older adults are more likely to develop inappropriate prescriptions [42]. Multiple medication use is the strongest risk factor for various drug-related issues and the strongest predictor of renal inappropriate prescription [43]. Combination of potential nephrotoxic drugs may have induced the accelerated deterioration in renal function [27]. Therefore, medical staff should pay attention to the possibility that elderly patients may experience more diverse reactions when taking multiple medications [37]. Understanding the pharmacokinetics of drugs in the elderly and the interactions between drugs is necessary, as it not only helps clinical staff make appropriate dosing decisions for patients, but also helps reduce the damage of drugs to kidney disease.

In addition, we also found that elevated blood pressure, blood urea nitrogen, triglycerides, and white blood cell levels were all independent risk factors for CKD in the elderly. Long-term high blood pressure damages the kidneys by causing renal intimal thickening, lumen stenosis, and insufficient renal blood supply [44]. And elevated TG have been established as well-known traditional risk factors for CKD, which also has been confirmed in other studies [45, 46]. White blood cells were often considered a traditional indicator of infection, inflammation, and disease progression, closely related to the occurrence and development of CKD. And higher BUN levels have also been identified as a risk factor for renal disease progression in patients with moderate to severe CKD [47]. When abnormalities in these indicators may also require patients to take more medication for treatment, increasing the risk of patients taking multiple medications. Therefore, the physical condition of elderly patients should be evaluated from multiple aspects, awareness of the risks of multiple drug treatments should be raised, and appropriate intervention measures should be actively provided to reduce kidney damage.

## Strengths and limitations

Strengths of this study include the following: (1) the large sample of this study was from 18 community health service stations, and the data collected by well-trained teams was representative. (2) the medications were summarized using a standardized classification system to make the results more reliable and compared with other studies. (3) we adjusted for important confounding factors affecting renal function such as age, BMI, comorbidities and biochemical indicators to explore the relationship between the number of medications and CKD. However, our study also has some limitations. Firstly, the scarcity of similar studies in the literature limited the direct comparisons. Second, memory bias caused by self-reported medications could not be excluded, and the population taking 6, 7, and 8 drugs were relatively small, which might lead to the underestimation of polypharmacy. Finally, although we adjusted for several potential confounders, those unmeasured or residual confounders may still affect our findings.

## Conclusion

In the community, comorbidities and the use of multiple medications were common for elderly people. Our study revealed that as the number of medications taken increases, the risk of CKD was increasing. Our results were of great significance in promoting rational drug use and preventing the impact of multiple drug use on renal function in older people, which is increasingly in line with the global awareness of strengthening health systems and combating inappropriate medication use.

### Electronic supplementary material

Below is the link to the electronic supplementary material.

Supplementary Material 1

## Data Availability

The dataset used during the current study is available from the corresponding author on reasonable request.
