# Supplementary material for: Association between polypharmacy and chronic kidney disease among community-dwelling older people: a longitudinal study in southern China
Source: BMC Nephrol. 2024 May 17;25:169. doi: 10.1186/s12882-024-03606-x (PMC11100214; doi:10.1186/s12882-024-03606-x)
Supplement: Supplementary file 1 — Supplementary Material 1 [file 12882_2024_3606_MOESM1_ESM.docx]

Supplementary materials

Supplementary Table 1 The relationship between CKD and number of medications in logistic regression model

| Variable | B | $S_{\overline{x}}$ | Waldχ^2^ | OR 95%CI | *P* |
| --- | --- | --- | --- | --- | --- |
| Age  Gender (female)  Comorbidities (Yes)  SBP (mmHg)  DBP (mmHg)  WC (cm)  BMI (kg/m2)  WBC (109/L)  TG (mmol/L)  LDL-C (mmol/L)  BUN (mmol/L)  TBIL (μmol/L)  N. of medication  0  1  2  3  4  5  6  7  8 | 0.106  -0.220  -0.227  0.012  0.001  0.001  0.049  0.103  0.099  -0.092  0.078  -0.007  0.408  0.461  0.528  0.483  1.194  2.089  1.205  2.557 | 0.012  0.119  0.137  0.005  0.008  0.011  0.028  0.030  0.034  0.069  0.024  0.010  0.160  0.156  0.186  0.226  0.361  1.072  0.951  1.427 | 86.673  3.401  2.732  7.466  0.005  0.005  2.993  11.672  8.450  1.766  11.017  0.500  26.093  6.531  8.619  7.973  4.571  10.851  3.825  1.566  3.193 | 1.113(1.088-1.138)  0.806(0.638-1.018)  0.800(0.611-1.046)  1.013(1.004-1.022)  1.001(0.985-1.016)  1.001(0.979-1.023)  1.050(0.994-1.110)  1.109(1.046-1.177)  1.107(1.036-1.183)  0.912(0.796-1.045)  1.082(1.033-1.133)  0.993(0.974-1.012)  Reference  1.507(1.100-2.064)  1.582(1.165-2.149)  1.691(1.174-2.436)  1.625(1.043-2.532)  3.281(1.618-6.653)  8.130(0.995-66.400)  3.289(0.510-21.231)  12.802(0.781-209.767) | <0.001  0.070  0.103  0.006  0.946  0.948  0.083  0.001  0.003  0.185  0.001  480  0.001  0.011  0.003  0.005  0.032  0.001  0.051  0.211  0.074 |

Adjusted for age, gender, SBP, DBP, WC, BMI, WBC, BUN, TG, LDL-C, comorbidities. Abbreviations: SBP systolic blood pressure, DBP diastolic blood pressure, WC waist circumference, BMI body mass index, WBC: white blood cell, TG: triglyceride, LDL-C: low-density lipoprotein cholesterol, BUN: blood urea nitrogen.
